# Supplementary material for: Productive Performance, Physiological Variables, and Carcass Quality of Finishing Pigs Supplemented with Ferulic Acid and Grape Pomace under Heat Stress Conditions
Source: Animals (Basel). 2023 Jul 24;13(14):2396. doi: 10.3390/ani13142396 (PMC10376859; doi:10.3390/ani13142396)
Supplement: Supplementary file 1 [file animals-13-02396-s001.zip › animals-2503315-supplementary.pdf]

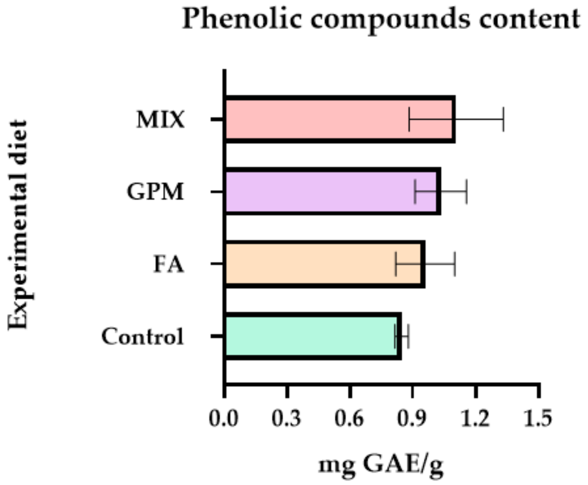

Supplementary Figure S1. Phenolic compounds content in experimental diets. Control (basal diet, BD without additives); FA, BD + 25 mg FA/ kg feed; GPM, BD + 2.5% GPM/ kg; and MIX, BD + 25 mg FA + 2.5% GPM/kg. GAE: Gallic acid equivalents. Bars are the mean ±standard error of 3 replicates.

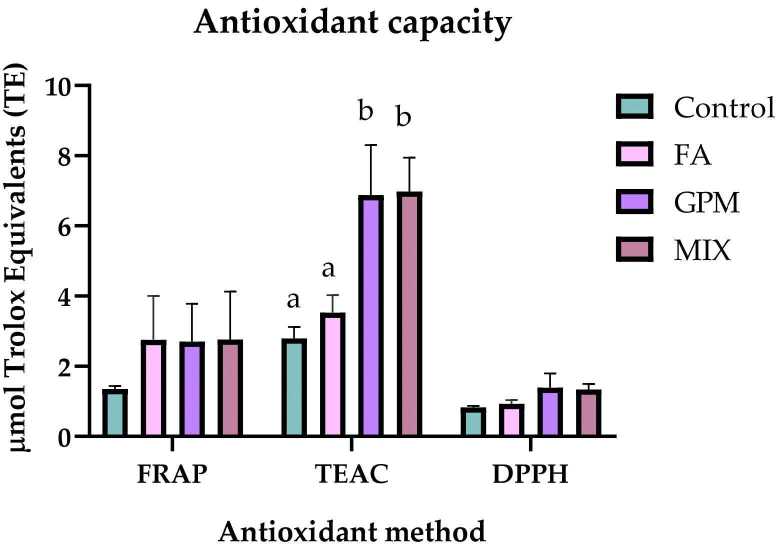

Supplementary Figure S2. Antioxidant capacity of experimental diets. Control (basal diet, BD without additives); FA, BD + 25 mg FA/ kg feed; GPM, BD + 2.5% GPM/ kg; and MIX, BD + 25 mg FA + 2.5% GPM/kg. Bars are the mean ±standard error of 3 replicates.
